# MitoQ Protects Ovarian Organoids against Oxidative Stress during Oogenesis and Folliculogenesis In Vitro

**DOI:** 10.3390/ijms24020924

**Published:** 2023-01-04

**Authors:** Jiapeng Wang, Hua Du, Lixin Ma, Mingqian Feng, Liping Li, Xiaorong Zhao, Yanfeng Dai

**Affiliations:** 1 College of Life Sciences, Inner Mongolia University, Hohhot 010070, China; 2 College of Life Sciences and Technology, Inner Mongolia Normal University, Hohhot 010022, China

**Keywords:** ovarian organoid, MitoQ, in vitro-generated oocyte, female germline stem cell, granulosa cell

## Abstract

Ovarian organoids, based on mouse female germline stem cells (FGSCs), have great value in basic research and are a vast prospect in pre-clinical drug screening due to their properties, but the competency of these in vitro-generated oocytes was generally low, especially, in vitro maturation (IVM) rate. Recently, it has been demonstrated that the 3D microenvironment triggers mitochondrial dysfunction during follicle growth in vitro. Therefore, therapies that protect mitochondria and enhance their function in oocytes warrant investigation. Here, we reported that exposure to 100 nM MitoQ promoted follicle growth and maturation in vitro, accompanied by scavenging ROS, reduced oxidative injury, and restored mitochondrial membrane potential in oocytes. Mechanistically, using mice granulosa cells (GCs) as a cellular model, it was shown that MitoQ protects GCs against H_2_O_2_-induced apoptosis by inhibiting the oxidative stress pathway. Together, these results reveal that MitoQ reduces oxidative stress in ovarian follicles via its antioxidative action, thereby protecting oocytes and granulosa cells and providing an efficient way to improve the quality of in vitro-generated oocytes.

## 1. Introduction

Gametes are tasked with faithfully transmitting genetic and epigenetic information from one generation to the next. Oocytes, as female germ cells, provide the necessary maternally derived organelles support for the development of embryos, especially mitochondria. Generally, ovarian reserve is established early in life with a finite number of oocytes. Currently, there is no direct evidence that adult oogenesis ever occurs under physiological conditions [1]. Therefore, the regeneration of oocytes is one of the most important aims of researchers in the field of regenerative medicine. Concomitant with technological development in three-dimensional (3D) and organoid culture, the concept of oogenesis in vitro became a reality. Reconstitution ovary or ovarian organoid, derived from mouse pluripotent stem cells (PSCs) [2] and FGSCs [3], respectively, have been successfully implemented to achieve the entire process of oogenesis in vitro. However, the competency of these in vitro-generated oocytes was markedly lower than that of in vivo-grown oocytes, and only a few of them developed to term after in vitro maturation (IVM) and in vitro fertilization (IVF) [2].

Takashima et al. [4] indicated that mouse follicles in vitro culture may be subjected to oxidative stress under conventional 3D culture conditions, which, in turn, leads to mitochondrial dysfunction in oocytes. ROS, a crucial factor in oocyte maturation, is produced in a healthy follicle under normal physiological activities [5]. While excessive ROS levels can cause mitochondrial dysfunction, thereby affecting oocyte maturation and subsequent fertilization [6]. Due to paternal mitochondria being damaged after sperm enters the oocyte, the quality of maternal mitochondria is vital for proper embryonic development [7]. Therefore, how to improve the in vitro gametogenesis environment and reduce the oxidative stress level are essential for the acquisition of oocyte competence.

MitoQ (MitoQuinone), as a derivative of ubiquinone conjugated to triphenylphosphonium, bearing a lipophilic cation, can enter and accumulate within the mitochondria as a result of the electrochemical gradient [8,9,10]. As a consequence of MitoQ’s ability to maintain mitochondrial function by inhibiting intracellular and mitochondrial ROS production and normalizing mitochondrial membrane potential (ΔѰm) has been tested in clinical trials for many diseases [11,12,13]. A long-term MitoQ treatment did not affect the total level of gene expression in mammalian tissues [14], suggesting that long-term administration of MitoQ to animals is safe and that, in vivo, it accumulates within mitochondria at appropriate concentrations to exert protection. Previous studies reported that MitoQ treatment during IVM in human, mouse [15], and porcine [16] oocytes can significantly improve the maturation rates and oocyte quality, which together indicate their potential for application in the ovarian organoid culture.

In the present study, the antioxidant effect of MitoQ is investigated during oogenesis in vitro culture. The treatment performance of MitoQ to mouse ovarian organoids is demonstrated by follicles size, maturation rate, ROS production, mitochondrial content, and ΔѰm in in vitro-generated oocytes. Meanwhile, the mechanisms of MitoQ against H_2_O_2_-induced apoptosis in GCs have been revealed. Our work provides an effective strategy for treating reproductive defects caused by 3D environmental stress.

## 2. Results

### 2.1. MitoQ Enhanced the Maturation of In Vitro-Generated Oocytes

**Figure 1:**
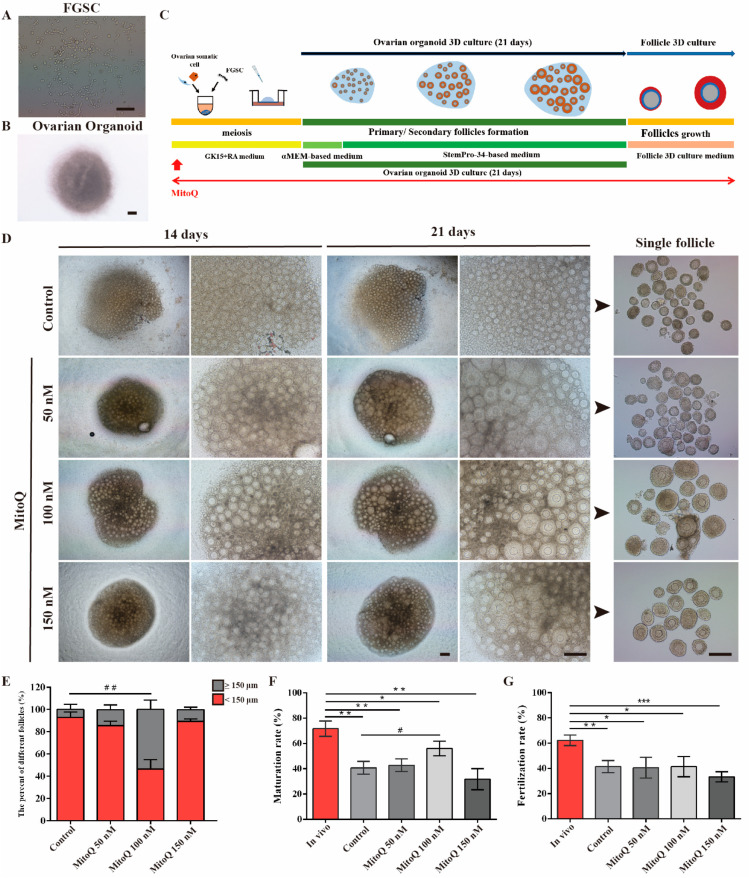
MitoQ promotes follicle growth and maturation in vitro. (**A**) FGSCs colony morphology. Scale bar, 200 μm. (**B**) Representative images of ovarian organoids at day 3. Scale bar, 100 μm. (**C**) Schematic diagram of the generation of ovarian organoids. (**D**) Representative images of organoids differentiated from FGSCs at 14 and 21 days of culture (Left). Scale bar, 200 μm. Representative images of single follicles isolated from ovarian organoids (right). Scale bar, 200 μm. (**E**) Quantification analyses of different sizes follicles (*n* = 3), error bars indicate SD. ^##^ *p* < 0.01, vs. control group. (**F**) Quantification analyses of IVM rate (*n* = 3). In vivo: in vivo-derived oocytes. Error bars indicate SD. * *p* < 0.05, ** *p* < 0.01, vs. in vivo group, ^#^ *p* < 0.05, vs. control group. (**G**) Quantification analyses of fertilization rate (*n* = 3). In vivo: in vivo-derived oocytes. Error bars indicate SD. * *p* < 0.05, ** *p* < 0.01, *** *p* < 0.001, vs. in vivo group.

To establish FGSC lines, FGSCs were isolated and cultured as in the previous study [17]. After sorting and culturing for 48 h, numerous FGSC clusters could be seen (Figure 1A). Next, FGSCs were aggregated with ovarian somatic cells to produce ovarian organoids and were subject to 3D culture (Figure 1B). The experimentation timeline and the route of 3D ovarian organoid generation and culture are illustrated in Figure 1C. To explore the effect of MitoQ during oogenesis in vitro, ovarian organoids were cultured in fresh medium supplemented with different concentrations of MitoQ (50, 100, 150 nM). Ovarian organoids without the addition of MitoQ served as the control group. During the whole culture process, FGSCs differentiated and eventually formed the complete follicles after 21 days of culture (Figure 1D). Then, the primary/secondary follicles were immediately isolated from the ovarian organoid and subjected to count the number of follicles based on follicle diameter (≥150 and <150 μm). At the MitoQ concentration of 50 nM and 150 nM, compared with the control side, the proportion of ≥150 μm follicles was not compromised (Control: 7.04% ± 4.79 vs. MitoQ 50 nM 14.54% ± 4.01, *p* > 0.05; vs. MitoQ 150 nM: 10.65% ± 2.12, *p* > 0.05, Figure 1F). Notably, when the concentration reached up to 100 nM, GCs exhibited robust growth and formed the rich external layer of oocytes. There was a significant rise in the proportion of ≥150 μm follicles by 53.54% ± 4.51 (*p* < 0.01, Figure 1E) in comparison with the control group.

Then, individual follicles were subjected to in vitro growth. After 11 days of culture, in vitro-generated (IVG) oocytes grew into GV oocytes among the experimental groups. When transferring them under the in vitro maturation (IVM) culture conditions, 40.75% of the GV oocytes extruded a first polar body in the control group. As the MitoQ concentration rose, the maturation rate of IVG oocytes began to rise gradually. The highest maturation rate was obtained when the concentration of MitoQ reached 100 nM, compared to the control side (MitoQ 100 nM: 56.00% ± 5.83 vs. control: 40.75% ± 5.10, *p* < 0.05, Figure 1F). Here, it should be remarked that, despite the maturation rate of IVG oocytes being ameliorated with MitoQ treatment, it remained significantly lower than in the in vivo-grown (in vivo) oocytes. Next, we conducted in vitro fertilization experiments to confirm the developmental competence of these mature oocytes. The fertilization rates of mature oocytes derived from organoids with MitoQ treatment did not differ significantly from those of controls (*p* > 0.05, Figure 1G). It is also noteworthy that the fertilization rates in the IVG group were markedly lower than those in the In vivo oocytes. Based on these results, MitoQ treatment has a positive effect on IVG oocyte growth and maturation.

### 2.2. Reversal of ROS-Mediated Mitochondrial Defects in Oocytes by MitoQ

**Figure 2:**
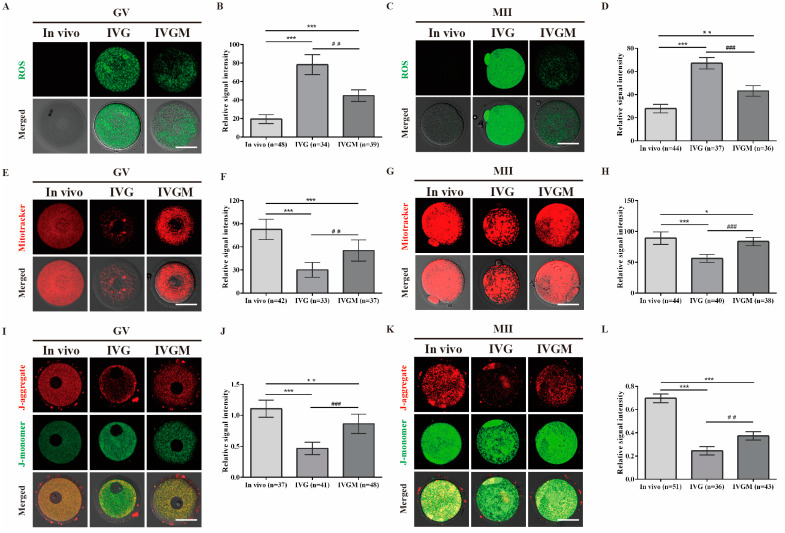
MitoQ improved mitochondrial function in in vitro-generated oocytes. (**A**,**C**) DCFH-DA staining of GV and MII oocytes, scale bar, 50 μm. In vivo: in vivo-derived oocytes; IVG: in vitro-generated oocytes without MitoQ treatment; IVGM: in vitro-generated oocytes with 100 nM MitoQ treatment. (**B**,**D**) Relative fluorescence intensity of DCFH-DA staining in GV and MII oocytes. Error bars indicate SD. ** *p* < 0.01, *** *p* < 0.001, vs. in vivo group, ^##^ *p* < 0.01, ^###^ *p* < 0.001, vs. IVG group. (**E**,**G**) MitoTracker staining of GV and MII oocytes, scale bar, 50 μm. (**F**,**H**) Relative fluorescence intensity of MitoTracker staining in GV and MII oocytes. Error bars indicate SD. * *p* < 0.05, *** *p* < 0.001, vs. in vivo group, ^##^ *p* < 0.01, ^###^ *p* < 0.001, vs. IVG group. (**I**,**K**) JC-1 staining of GV and MII oocytes, scale bar, 50 μm. (**J**,**L**) The ratio of red to green fluorescence intensity in GV and MII oocytes. Error bars indicate SD. ** *p* < 0.01, *** *p* < 0.001, vs. in vivo group, ^##^ *p* < 0.01, ^###^ *p* < 0.001, vs. IVG group.

To further investigate the mechanism by which MitoQ improved IVG oocyte maturation, we used the probe DCFH-DA to detect ROS levels in in vivo and IVG oocytes at GV and MII stages. As shown in Figure 2A,B, at the GV stages, ROS levels were significantly higher in IVG oocytes compared to the in vivo oocytes. These differences became more critical at the MII stage as the ROS levels increased in IVG oocytes (*p* < 0.001, Figure 2C,D), suggesting that 3D environment exposure increases the levels of intracellular ROS in IVG oocytes. With the addition of MitoQ during the organoid culture process (IVGM), ROS production was significantly decreased in GV and MII IVGM oocytes (GV, *p* < 0.001, Figure 2B; MII, *p* < 0.001, Figure 2D).

Generally, excessive ROS generation leads to mitochondrial damage that may perturb the mitochondria distribution and ΔѰm. Here, mitochondria distribution and ΔѰm levels were detected between in vivo and IVG oocytes at GV and MII stages. As indicated in Figure 2E,G, mitochondria were normally distributed throughout the cytoplasm in the in vivo group, while aggregated and clustered mitochondria were observed in the IVG groups. In addition, at the GV and MII stages, the quantitative analyses of MitoTracker results showed that the fluorescence intensity was significantly decreased in IVG oocytes compared to the in vivo oocytes (GV, *p* < 0.001, Figure 2F; MII, *p* < 0.001, Figure 2H). With the inclusion of MitoQ during organoid culture, the fluorescence signals of mitochondria were significantly upregulated (GV, *p* < 0.01, Figure 2F; MII, *p* < 0.001, Figure 2H) compared with that of the IVG group. Next, we assessed changes of ΔѰm using the JC-1 probe and measured the red/green ratio. In comparison to in vivo GV oocytes, a decrease in ΔѰm in IVG oocytes was observed (*p* < 0.001, Figure 2I,J). However, after treatment of MitoQ, ΔѰm was significantly increased in IVGM oocytes compared with the IVG oocytes (*p* < 0.001, Figure 2I,J). The differences in the MII stage between these groups also showed the same pattern (Figure 2K,L). These data indicate that MitoQ can improve mitochondrial quantity in IVG oocytes.

### 2.3. MitoQ Promoted GCs Proliferation

**Figure 3:**
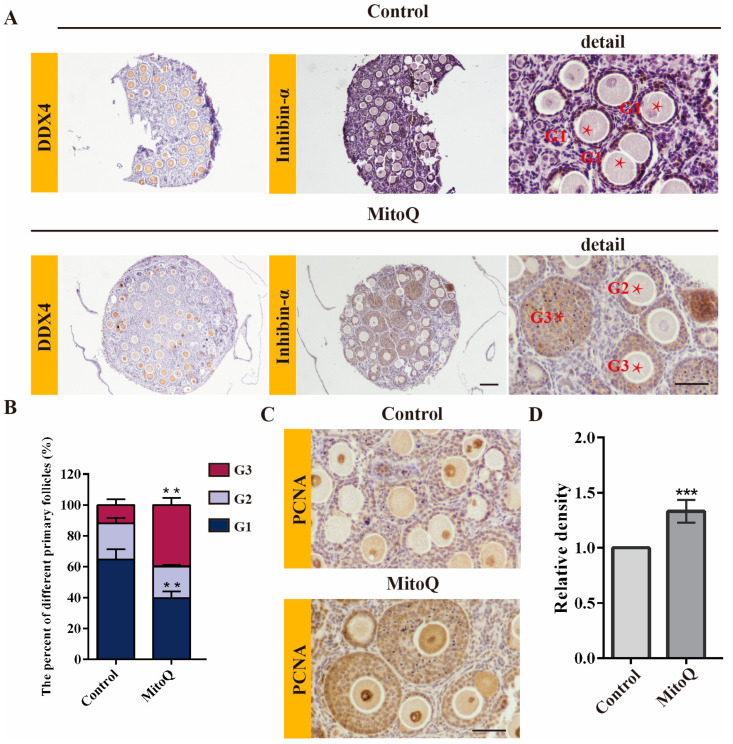
MitoQ promotes the proliferation of GCs during oogenesis in vitro. (**A**) Immunohistochemical detection of DDX4 and INHIBIN-α in ovarian organoids after 21 days of culture. Scale bar, 100 μm (left), 50 μm (right). (**B**) The percentage of different grade follicles on the organoid (*n* = 3). Grade 1 (G1): an oocyte surrounded by a monolayer of GCs; Grade 2 (G2): an oocyte surrounded with two layers of GCs; Grade 3 (G3): like Grade 2, but with more than three layers of GCs with the larger size. Error bars indicate SD. ** *p* < 0.01, vs. control group. (**C**) Immunohistochemical detection of PCNA in ovarian organoids (5 sections per organoid, *n* = 3). Scale bar, 50 μm. (**D**) The relative intensity of PCNA. Error bars indicate SD. *** *p* < 0.001, vs. control group. All sections were 3–5 μm serial sections.

The above findings indicated that MitoQ could improve follicles growth and increase follicle size during folliculogenesis in vitro, which seems to indicate that MitoQ contributes to GCs proliferation. Here, we assessed the number and morphology of follicles in ovarian organoids after 21 days of culture. The spatiotemporal profiles of DDX4 (oocytes) and INHIBIN-α (GCs) were examined by immunohistochemical staining (Figure 3A). Compared with the control side, the proportion of G3 follicles was significantly higher in MitoQ-treated ovarian organoids (MitoQ: 39.89% ± 4.52 vs. control: 11.91% ±3.69, *p* < 0.01, Figure 3B). To characterize the follicles growth at the cellular level, we examined the proliferative rate of GCs in developing follicles based on PCNA incorporation staining. Accordingly, based on regions where PCNA staining coincided, GCs of MitoQ-treated ovarian organoids demonstrated significantly increased levels of proliferation (*p* < 0.001, Figure 3C,D). The results indicate that MitoQ enhances the proliferation of surrounding GCs, which, in turn, might contribute to the improvement of follicular development.

### 2.4. MitoQ Inhibits Mitochondria Injury in H_2_O_2_-Treated GCs

**Figure 4:**
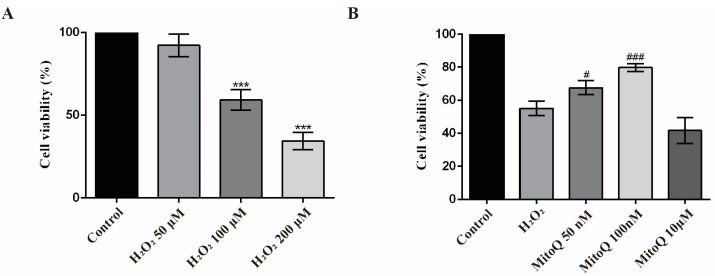
Effect of MitoQ treatment on cell viability in H_2_O_2_-stimulated GCs. (**A**) Quantification analyses from CCK-8 assays to detect cell viability of GCs treated with different concentrations of H_2_O_2_ for 24 h (*n* = 3). Error bars indicate SD. *** *p* < 0.001, vs. control group. (**B**) Quantification analyses from CCK-8 assays to detect cell viability of GCs treated with different concentrations of MitoQ for 24 h after H_2_O_2_ exposure (*n* = 3). Error bars indicate SD. ^#^ *p* < 0.05, ^###^ *p* < 0.001, vs. H_2_O_2_ group.

Follicular GCs are closely related to ovarian function. Here, we constructed an H_2_O_2_-treated GCs oxidative stress model and used the CCK8 array to assess the effects of MitoQ on GCs. The untreated control (no H_2_O_2_ and no MitoQ) was set as 100%. CCK8 results indicated that various H_2_O_2_ exposure (50, 100, and 200 μM) showed concentration-dependent inhibition of GCs survival. When cells were treated with 100 μM H_2_O_2_ for 24 h, the cell survival rate was 59.17% ± 6.20, which was closest to the IC50 (Figure 4A). Therefore, 100 μM was selected for subsequent experiments as the optimal concentration. Next, we investigated whether MitoQ reversed the cell viability change induced by H_2_O_2_. As shown in Figure 4B, the loss of GCs viability upon H_2_O_2_ exposure was partially rescued in GCs pretreated with MitoQ (50 nM, *p* < 0.05). When even higher MitoQ concentrations (100 nM) were achieved, the viability defect in GCs was significantly relieved (*p* < 0.01) compared with the H_2_O_2_ group.

**Figure 5:**
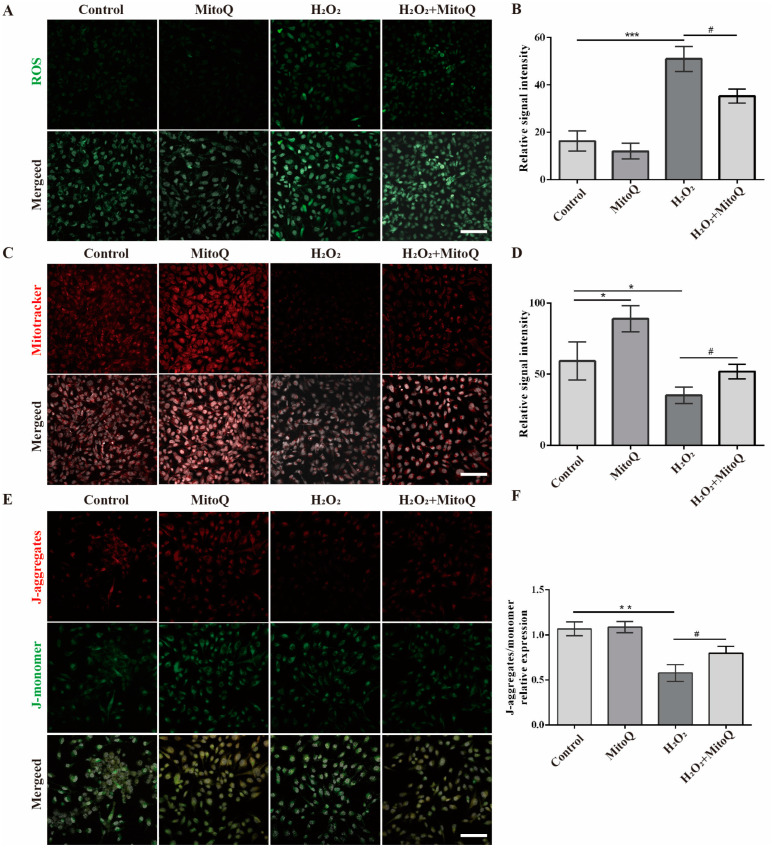
MitoQ protects the mitochondria in GCs from H_2_O_2_-induced oxidative stress. (**A**) DCFH-DA staining of GCs, DNA (DAPI) is shown in gray, scale bar, 100 μm. (**B**) Relative fluorescence intensity of DCFH-DA staining in GCs (*n* = 3). Error bars indicate SD. *** *p* < 0.001, vs. control group. ^#^ *p* < 0.05, vs. H_2_O_2_ group. (**C**) MitoTracker staining of GCs, DNA (DAPI) is shown in gray. Scale bar, 100 μm. (**D**) Relative fluorescence intensity of MitoTracker staining in GCs (*n* = 3). Error bars indicate SD. * *p* < 0.05, vs. control group. ^#^ *p* < 0.05, vs. H_2_O_2_ group. (**E**) JC-1 staining of GCs, DNA (DAPI) is shown in gray. Scale bar, 100 μm. (**F**) The ratio of red to green fluorescence intensity in GCs (*n* = 3). Error bars indicate SD. ** *p* < 0.01, vs. control group. ^#^ *p* < 0.05, vs. H_2_O_2_ group.

Then, we further evaluate the antioxidant of MitoQ on GCs in several aspects, including ROS production, Mitochondrial distribution, and ΔѰm. Using DCFH-DA staining, we observed that the intracellular ROS production of H_2_O_2_-treated GCs was higher than those in the control group (*p* < 0.001, Figure 5A,B), but the effect was obviously reversed after treatment of MitoQ (*p* < 0.05, Figure 5A,B). To further evaluate the mitochondrial damage, we next stained GCs with MitoTracker and JC-1. As indicated in Figure 4E, the result showed a significant reduction of functional mitochondria in GCs with H_2_O_2_ treatment versus controls (Figure 5C,D, *p* < 0.05), while MitoQ produced a significant mitigating effect on mitochondrial depolarization during oxidative stress (*p* < 0.05, Figure 5C,D). In parallel, the staining of JC-1 results showed that H_2_O_2_ treatment increased the fraction of depolarized mitochondria (*p* < 0.01, Figure 5E,F), which was significantly reversed by MitoQ treatment (*p* < 0.05, Figure 5E,F). Together, these results proved that MitoQ pretreatment effectively decreased the mitochondrial oxidative stress in GCs and contributed to the protection.

### 2.5. MitoQ Suppresses the Apoptosis Caused by Oxidative Stress

**Figure 6:**
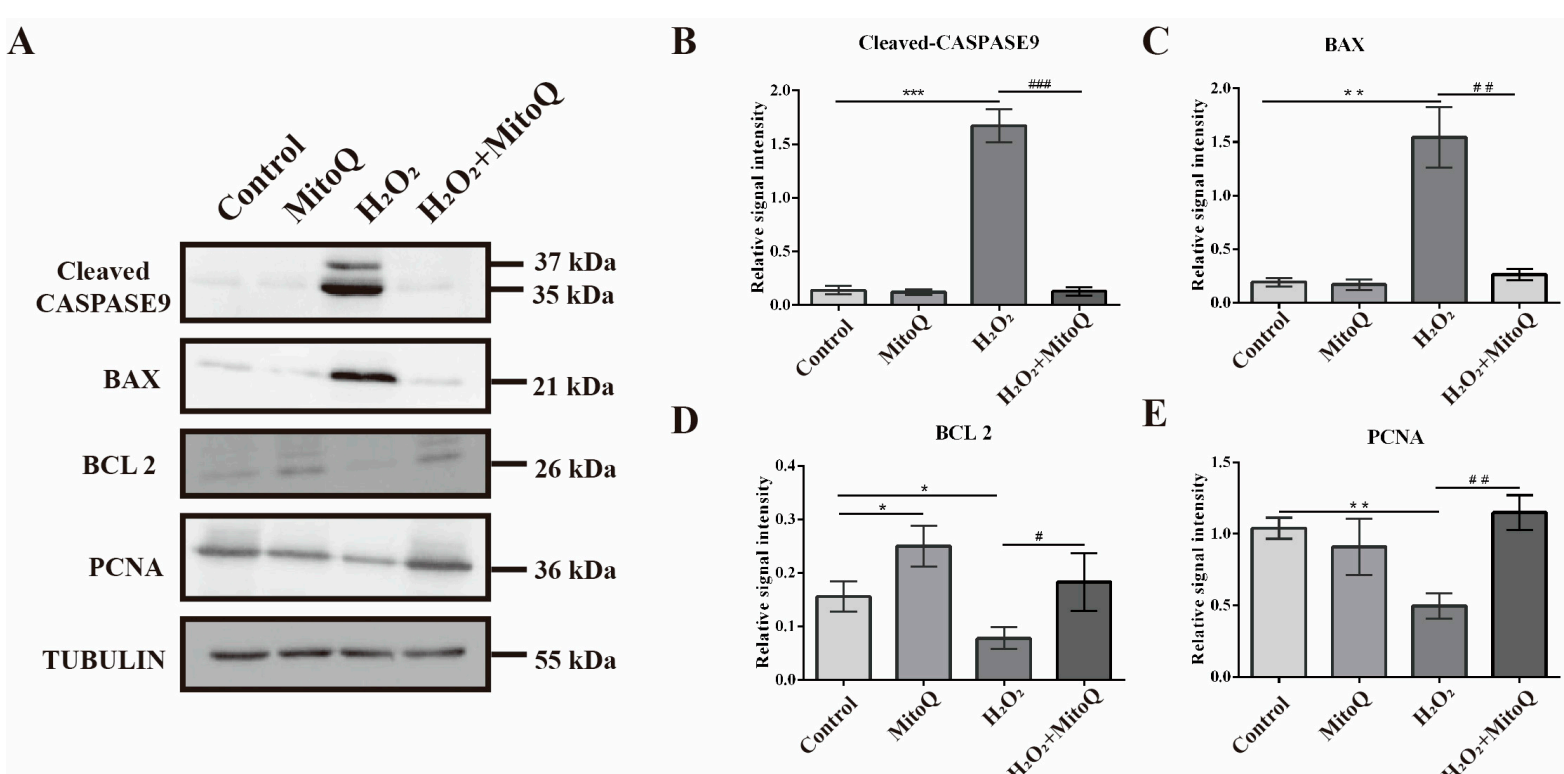
MitoQ can prevent cell damage induced by H_2_O_2_-induced apoptosis. (**A**) CASPASE 9, BAX, BCL 2, and PCNA protein expression detected by Western blot (*n* = 3). TUBULIN was used as a loading control. (**B**–**E**) Quantification of Western blot results. Error bars indicate SD. * *p* < 0.05, ** *p* < 0.01, *** *p* < 0.001, vs. control group. ^#^ *p* < 0.05, ^##^ *p* < 0.01, ^###^ *p* < 0.001, vs. H_2_O_2_ group.

It was found that the dysfunction of mitochondria often resulted in cell death, and some mitochondrial apoptosis-related genes, such as caspase family and apoptosis-related genes, were often regulated. Consistent with this, our results showed that exposure to H_2_O_2_ alone caused increased expression level of CASPASE 9 (*p* < 0.001, Figure 6A,B), BAX (*p* < 0.01, Figure 6A,C) and decreased the expression of BCL 2 (*p* < 0.05, Figure 6A,D) and PCNA (*p* < 0.01, Figure 6A,E) in GCs, in comparison to control group. Apparently, these changes were alleviated by the administration of MitoQ when compared to the H_2_O_2_-treated group. Collectively, our results revealed that MitoQ was able to greatly attenuate cell death induced by oxidative stress.

## 3. Discussion

Ovarian organoids are an emerging culture technology worth pursuing many applications. The ability of ovarian organoids to be monitored and manipulated in a controlled environment makes them an ideal model for exploring the potential mechanisms of oogenesis [18]. It also provides a tractable system for pre-clinic medicines screening [3]. At present, both PSCs and FGSC have been used to produce ovarian organoids, and these resultant oocytes have produced healthy pups [2,3]. Clearly, ovarian organoids have shown great potential in biomedical applications owing to their uniform characteristics. Nevertheless, the question remains as to the identification of the effective culture condition that permits both efficient and consistent production of competent oocytes during reconstitution culture. In this study, we investigated the role of MitoQ in the recovery of oocyte competency and identified the pathway impacting oocyte competency and GCs proliferation by molecular analyses.

Numerous biochemical processes are performed by mitochondria, including the production of ATP, the regulation of Ca^2+^ levels, and the synthesis of steroid hormones [19]. The maintenance of mitochondria within the germline is a highly regulated process. At an early embryonic developmental stage in mice (E9.5 to E11.5), each PGC contains less than 1000 mitochondria [20]. Just prior to primordial follicle formation, mitochondria are transported from oocytes in the nest to one (or a small number) of oocytes in each nest. At this point, the content of mitochondria does not increase [21]. During and immediately after birth, the ovary is populated by primordial oocytes, which each contain about 5000 mitochondria [22]. Thus, to a somewhat lesser degree, the contact with the development of the follicles and the mitochondria number was interrelated. Normally, ROS are produced by oocyte metabolism under normal physiological conditions, but when ROS levels exceed physiological levels, mitochondria become unstable, resulting in mitochondrial damage. The impaired mitochondrial function causes a range of defects, including the integrity disruption of the meiotic and mitotic spindles, thereby disrupting oocyte maturation and embryo development [23,24,25].

Antioxidants are compounds that can delay or inhibit oxidation from reacting with other molecules by the transfer of electrons, thereby maintaining cellular redox homeostasis and proteostasis. In a broad sense, antioxidants can be divided into two broad categories: endogenous and exogenous [26]. The intracellular endogenous antioxidant defense system comprises enzymatic antioxidants (SODs GPx and catalase) along with some nonenzymatic antioxidants (vitamin E, glutathione, and bilirubin) [27,28]. Exogenous antioxidants include carotenoids [29], flavonoids (e.g., anthocyanin) [30], and vitamins (e.g., A/C) [31,32,33]. Endogenous and exogenous antioxidants coordinately regulate the maintenance of mitochondria function and, in doing so, helps to protect cells from oxidative stress-induced damage, thus ensuring their proliferation and survival. Mechanistically, different antioxidants are differentially distributed in spaces, with the most distributed within the cytoplasm (untargeted antioxidants) and a small number localized within the mitochondria (Mt-targeted antioxidants) [28,34,35,36]. The mechanism of action and protective effects of untargeted and Mt-targeted antioxidants has been extensively studied. Compared with untargeted cellular antioxidants, the Mt-targeted antioxidants exerts greater protection against oxidative damage in the mitochondria and eliminate ROS at the heart of the source through their ability to cross the mitochondria–phospholipid bilayer [34,35,36,37,38,39], such as MitoQ, which could be effective antioxidant therapies against the damage caused by enhanced ROS generation [12,36,40,41].

Currently, both for mice and humans, a previous study suggested that MitoQ protects oocytes against functional defects caused by ROS accumulation and aging [15]. In this study, the findings proved that MitoQ administration promoted follicle growth and oocyte maturation. Compared with in vivo oocytes, in vitro-generated oocytes exhibit higher basal ROS levels, abnormal mitochondrial distribution, less matured mitochondria, and lower ΔѰm; however, after the treatment of MitoQ, these changes could significantly be reversed to a certain extent. Collectively, these findings revealed the antioxidant effect of MitoQ during oogenesis in vitro on the one hand and indicated that oxidative stress induced by traditional 3D culture environment may be responsible for the low competency of in vitro-generated oocytes on the other hand.

The mammalian oocyte growth and maturation in a mutually dependent relationship with adjacent somatic cells. Due to the naive mitochondrial state of immature oocytes, the growth and maturation of immature oocytes are mostly supported by the surrounding GCs [42]. Accordingly, GCs have been shown to play a central role in providing metabolic support via gap junctional communication and maintaining adequate oocyte ATP levels [43]. It has been found that GCs-enclosed oocytes matured in vitro have higher ATP contents throughout maturation than oocytes matured without their cumulus vestment [23]. In part, the observation of differing rates of follicles growth in vitro is likely to reflect oocyte quality. Differences in growth rates of immature follicles that survive during follicles in vitro culture have been reported in mice [44,45], caprine [46,47], cattle [48], and macaques [49,50]. Macaques, for instance, have only follicles that grow rapidly and are capable of forming mature oocytes and/or fertilized embryos [49]. In addition, faster-growing mice follicles also exhibited increased fertilization rates and a live birth [51]. In follicle development, the discrepancy in follicle growth may reflect the ability to synthesize and respond to autocrine/paracrine factors (AMH, GDF9, BMP15), which modulate GCs proliferation [52]. In the present experiment, our findings demonstrated that the size of the follicle with MitoQ treatment increased greatly during folliculogenesis in vitro, accompanied by a rapid proliferation of GCs, which indicated that MitoQ promotes the proliferation of GCs. Moreover, by the H_2_O_2_-oxidative injury model, we further validated that MitoQ guards GCs against the H_2_O_2_-induced apoptosis pathway (caspase 9 and Bax) via the preservation of mitochondrial function. Therefore, we have reason to believe that the beneficial effect of MitoQ on oocyte potential may be largely attributable to the GCs proliferation.

Aging is known to be associated with sub-optimal reproductive performance in females. However, the exact mechanisms by which it affects fertility are complex [53]. State-of-the-art research suggests mitochondrial dysfunction in GCs may play a key role in the age-related decline in ovarian function and reproductive capacity [54]. In studies, MitoQ, a derivative of coenzyme Q, effectively improves mitochondrial function [9] and attenuates redox-related diseases [55,56]. At present, for mice, humans [15], and porcine [16], multiple studies have shown that MitoQ promotes oocyte maturation in vitro and maintains the stability of mitochondrial function. Furthermore, a recent experimental study demonstrated that MitoQ is able to alleviate ovarian fibrosis in obese and reproductively old mice [57]. Our results revealed that MitoQ could protect GCs against oxidative stress, thereby improving in vitro-generated oocytes maturation competence. This suggests that the administration of MitoQ may be considered an intervention strategy for anti-ovarian aging therapy for humans.

## 4. Materials and Methods

### 4.1. Animal Breeding

The outbred ICR mouse was purchased from SPF Biotechnology (Beijing, China). Mice were bred in the mouse house of Inner Mongolia University in the standard temperature/humidity constant environment.

### 4.2. Chemicals

MitoQuinone (MitoQ, Cat. No. S8978) was obtained from Selleck (Washington, DC, USA).

### 4.3. FGSCs Extraction and Culture

In this study, protocols for FGSCs isolation and sorting were identical to previous studies [17,58,59]. In brief, ovaries from 1–3 dpp female mice were digested to the single cell suspension using a two-step enzymatic method (0.25% trypsin (Cat. No. 15050065, Gibco, Waltham, MA, USA) and 1 mg/mL collagenase (type IV, Cat. No. 17104019, Gibco)), and then the antibody against Fragilis (Cat. No. PA5-34598, Thermo Fisher Scientific, Waltham, MA, USA) and Dynabeads M-280 Sheep anti-Rabbit IgG (Cat. No. 11203D, Thermo Fisher Scientific) were used for FGSCs sorting. The FGSCs were cultured in MEM-α (Cat. No. 12561056, Thermo Fisher Scientific) containing 10% fetal bovine serum (Cat. No. 10099, Gibco), 2 mM Gluta^max^ (Cat. No. 35050061, Gibco), 1 mM Sodium Pyruvate (Cat. No. 11360070, Gibco), 1 mM MEM non-essential amino acids (Cat. No. 11140050, Gibco), 100 IU/mL Penicillin-streptomycin (Cat. No. 10378016, Gibco), 10 ng/mL recombinant Human FGF-basic (bFGF, Cat. No. 100-18B, PeproTech, London, UK), 10 ng/mL Human GDNF Recombinant Protein (RP-8602, Thermo Fisher Scientific), 20 ng/mL mouse epidermal growth factor (EGF, Cat. No. E5160, Sigma-Aldrich, St. Louis, MO), 10 ng/mL mouse leukemia inhibitory factor (LIF, Cat. No. LIF2010, Sigma-Aldrich), and β-mercaptoethanol (Cat. No. M6250, Sigma-Aldrich) at 37 °C with 5% CO_2_. The culture medium was changed every second day.

### 4.4. Three-Dimensional Culture

The ovarian organoid was formed using a modified method described elsewhere [2,3]. A brief description is given below, 1 × 10^3^ FGSCs co-culture with 3 × 10^4^ female gonadal somatic cells (from 1–3 dpp ovaries) in a U-bottomed 96-well plate for 2 d with GK15+RA medium (Glasgow MEM (Cat. No. 11710035, Thermo Fisher Scientific) supplemented with 15% Knockout serum replacement (Cat. No. 10828010, Thermo Fisher Scientific), 1.5 μM retinoic acid (RA, Cat. No. R2625-50MG, Sigma-Aldrich), 2 mM Gluta^max^, 1 mM MEM non-essential amino acids, 1 mM Sodium Pyruvate, 100 mM β-mercaptoethanol, 20 ng/mL EGF, 10 ng/mL bFGF, 10 ng/mL GDNF, 10 ng/mL LIF, and 100 IU/mL Penicillin-streptomycin). To extensively remove endogenous germ cells, gonadal somatic cells have been sorted by DDX4 (a universal marker of germ cells) antibodies conjugated with magnetic beads. Next, ovarian organoids were transferred onto Transwell-COL membranes (Cat. No. 3492, Corning, Corning, NY, USA) soaked in GK15+RA medium for 2 d and the medium was changed 24 h. Afterward, ovarian organoids cultured with α-MEM-based medium (α-MEM supplemented with 2% FBS, 2 mM Gluta^max^, 100 μM ascorbic acid (Cat. No. G0394, TCI, Tokyo, Japan), 20 ng/mL EGF, 50 mM β-mercaptoethanol and 100 IU/mL penicillin-streptomycin) and StemPro-34-based medium (StemPro34 SFM (Cat. No. 10639011, Gibco) supplemented with 10% FBS, 2 mM Gluta^max^, 100 μM ascorbic acid, 20 ng/mL EGF, 50 mM β-mercaptoethanol, 800 nM ICI182780, 100 IU/mL penicillin-streptomycin) for 21 d. The ovarian organoids were incubated at 37 °C with 5% CO_2_ and half the medium volume (0.6 mL) was changed every second day.

### 4.5. Follicle 3D Culture

Under a stereomicroscope, the individual follicles were isolated from ovarian organoids using 27-gauge needles and cultured with follicle 3D culture medium (α-MEM supplemented with 5% FBS, 2% polyvinylpyrrolidone (Cat. No. 9003-39-8, Sigma-Aldrich),2 mM Gluta^max^, 200 μM ascorbic acid, 50 mM β-mercaptoethanol, 100 IU/mL Penicillin-streptomycin, 20 ng/mL EGF, 1 mM Sodium Pyruvate, 0.2 IU/mL follicle-stimulating hormone (Cat. No. 2413405A1023, MSD, New Jersey, NJ, USA), 20 ng/mL BMP15 (Cat. No. 5096-BM-005, R&D, Santa Clara, CA, USA), and 20 ng/mL GDF9 (Cat. No. 739-G9-010, R&D) at 37 °C with 5% CO_2_. After 2 days of follicle 3D culture, follicles were treated with 0.1% type IV collagenase for 5 min, and further cultured in follicle 3D culture medium continued for 9 d, while changing the medium every other day. Following this, in vitro-grown follicles were subjected to IVM.

### 4.6. In Vitro Maturation and Fertilization

For in vitro maturation, cumulus oocyte complexes were maturated in vitro in a α-MEM based maturation media containing 5% FBS, 30 mg/mL sodium pyruvate, 0.1 IU/mL follicle-stimulating hormone, 4 ng/mL EGF, 1.2 IU/mL human chorionic gonadotrophin (Cat. No. 2413402X2053, ASKA, Tokyo, Japan), 4 ng/mL bFGF, 30 mg/ ml penicillin, 75 mg/mL streptomycin. After 16–18 h of maturation, MII oocytes were transferred into 90-µL human tubal fluid droplets (each containing 20–25 oocytes) covered with paraffin liquid in a 35-mm dish. Spermatozoa were collected from the cauda epididymis of adult ICR male mice and added at a final concentration of 2 × 10^6^ /mL to a drop of human tubal fluid medium containing the MII oocyte. After inoculation for 6 h at 37 °C, the oocytes were transferred to KSOM medium and further cultured for 18 h.

### 4.7. DCFH-DA, MitoTracker, and MitoProbe JC-Staining

The ROS production, distribution of mitochondria, and ΔѰm in oocytes were detected by ROS Assay Kit (Cat. No. S0033S, Beyotime, Nantong, China), MitoTracker Red CMXRos (Cat. No. C1049B, Beyotime), Enhanced mitochondrial membrane potential assay kit with JC-1 (Cat. No. C2003S, Beyotime), respectively. Briefly, GV/MII oocytes were cultured in DCFH-DA (10 μM), MitoTracker (200nM) and JC-1 (10 μM) solution for 30 min at 37 °C, respectively. After being washed three times in preheated culture medium, oocytes were transferred to the glass-bottom culture dish supplemented with PBS droplet and the signals from each group were detected with the same scanning settings immediately.

The level of ROS, mitochondria content and ΔѰm in GCs were detected as above. In brief, GCs were seeded on 6-well plates with a density of 8 × 10^4^ /well, after treatment with H_2_O_2_ or MitoQ according to the experimental design, cells were incubated with DCFH-DA (10 μM), MitoTracker (200 nM) and JC-1 (10 μM) solution for 30 min at 37 °C, respectively. After washing 3 times with PBS, 5 visual fields were randomly selected under the fluorescence microscope (Nikon, Tokyo, Japan) for observation. The fluorescence intensity of each sample was analyzed by Image J (U.S. National Institutes of Health, Bethesda, MD, USA, https://imagej.nih.gov/ij/, 1997–2018, accessed on 2 December 2020) software. ROS production and distribution of mitochondria were evaluated as the intensity of signals, respectively. ΔѰm was evaluated as the ratio of the red to green fluorescence intensity. Three samples from each group were assayed and performed in triplicate.

### 4.8. Immunohistochemical Staining

Ovarian organoids were fixed in 4% paraformaldehyde, embedded in paraffin and sectioned. For immunohistochemical analyses, the sections were dewaxed and rehydrated by xylol and alcohol, respectively. The section (3–5 μm) was furtherly immersed into 10 mM sodium citrate buffer, pH 6.0 and heated for 10 min. Then, the slides were treated with 1% H_2_O_2_ in PBS to inactivate endogenous peroxidase activity and incubated with blocking buffer, 10% serum in PBS, for 1 h at 37 °C. After that, the sections were incubated with the first antibody (DDX4: Cat. No. ab270534, Abcam, Cambridge, UK, 1:1000; Inhibin alpha: Cat. No. ab224798, Abcam, 1:1000, PCNA: Cat. No. 10205-2-AP, Proteintech, Wuhan, China, 1:500) overnight at 4 °C. The slides were washed three times in PBS, each time for 10 min, after which the slides were incubated with horseradish peroxidase (HRP) conjugated anti-rabbit IgG (Cat. No. A0279, Beyotime for 1 h at room temperature. HRP activity was detected with DAB solution (Cat. No. P0203, Beyotime. The slides were examined under a microscope and photos were taken for analysis by ImageJ. Staining was quantified by tracing the immunoreactive area (IA) and the integrated optical density (IOD). The staining intensity (SI) for each image was calculated as SI = IOD/IA. The follicles were classified according to their developmental stages based on the GCs morphology. All staining were repeated more than three times.

### 4.9. Granulosa Cells Culture

Primary GCs were isolated from PMSG-primed (24 h) PD23 female mice ovary according to described previously [60,61]. GCs were released from antral follicles by puncturing with a 27-gauge needle under a stereoscope. Cells were cultured at a density of 1 × 10^6^ cells in DMEM/F12 medium (Cat. No. 12634010, Gibco) containing 5% FBS, 6 mg/mL Penicillin-streptomycin in 24-well culture dishes. After overnight culture, cells were washed and cultured in serum-free medium before any further treatment.

### 4.10. CCK8 Array

GCs with 5 × 10^3^ cells per well were plated in 96-well plates. After 48 h of culture under DMEM/F12-based medium, GCs reached ~70% confluency. CCK8 reagent (Cat. No. C0038, Beyotime) was added to the 96-well plate (10 μL/well) and incubated accordingly. Finally, the absorption value was measured with a microplate reader (Bio-Tek Instruments, Thermo Fisher Scientific) at 450 nm wavelength. Three samples from each group were assayed, and each reaction was performed in triplicate.

### 4.11. Western Blot

Proteins of GCs were obtained by RIPA lysis buffer RIPA (Cat. No. P0013B, Beyotime) supplemented with a protease inhibitor cocktail in centrifuged tubes in ice for 20 min. The concentration of the protein was measured by using the BCA protein assay (Cat. No. 23225, Thermo Fisher Scientific). 30 μg of proteins from each sample were loaded to SDS-PAGE before transferring to nitrocellulose membranes (Bio-Rad Biotechnology, Hercules, CA, USA), and probed with the indicated antibodies (CASPASE 9: Cat. No. 10380-1-AP, Proteintech, 1:1000; BAX: Cat. No. 50599-2-Ig, Proteintech, 1:1000; BCL 2: Cat. No. 26593-1-AP, Proteintech, 1:1000; PCNA: Cat. No. 10205-2-AP, Proteintech, 1:1000; ALPHA TUBULIN: Cat. No. 11224-1-AP, Proteintech, 1:1000). Next day, the peroxidase-conjugated secondary antibody (Cat. No. SA00001-2, Proteintech, 1:2000) was added to incubate the mem-brane. Bands were visualized with the Clarity™ Western ECL Substrate (Cat. No. 32209, Thermo Fisher Scientific) and quantified with Image J. All samples were performed in triplicate.

### 4.12. Statistical Analysis

Experimental data were expressed as the means ± SD with each experiment. Analyses were conducted with SPSS (Version 24) and consisted of independent-samples *t*-test when only a single variable was manipulated or one-way ANOVA with Tukey–Kramer post hoc when multiple variables were manipulated. *p* < 0.05 was considered as a statistical significance.

## 5. Conclusions

In summary, our results demonstrated that MitoQ supplementation during ovarian organoids culture could reverse the 3D culture environment-induced oxidative stress by directly scavenging ROS and promoting the proliferation activity of GCs. The ameliorative influence of MitoQ was reflected by the improvement of mitochondrial function, follicle size, and ultimately by enhancement of oocyte maturation. To thoroughly explore the underlying mechanisms of MitoQ protection, further studies indicated that MitoQ alleviates oxidative stress and attenuates oxidative stress-associated damage in GCs. This study is warranted to validate that MitoQ may serve as a potential therapeutic drug for anti-ovarian aging.

## Data Availability

The data presented in this study are available on request from the corresponding author.

## References

[1] Zhang H., Liu L., Li X., Busayavalasa K., Shen Y., Hovatta O., Gustafsson J.A., Liu K. (2014). Life-long in vivo cell-lineage tracing shows that no oogenesis originates from putative germline stem cells in adult mice. Proc. Natl. Acad. Sci. USA.

[2] Hikabe O., Hamazaki N., Nagamatsu G., Obata Y., Hirao Y., Hamada N., Shimamoto S., Imamura T., Nakashima K., Saitou M. (2016). Reconstitution in vitro of the entire cycle of the mouse female germ line. Nature.

[3] Li X., Zheng M., Xu B., Li D., Shen Y., Nie Y., Ma L., Wu J. (2021). Generation of offspring-producing 3D ovarian organoids derived from female germline stem cells and their application in toxicological detection. Biomaterials.

[4] Takashima T., Fujimaru T., Obata Y. (2021). Effect of in vitro growth on mouse oocyte competency, mitochondria and transcriptome. Reproduction.

[5] Behrman H.R., Kodaman P.H., Preston S.L., Gao S. (2001). Oxidative stress and the ovary. J. Soc. Gynecol. Investig..

[6] Cecconi S., Ciccarelli C., Barberi M., Macchiarelli G., Canipari R. (2004). Granulosa cell-oocyte interactions. Eur. J. Obstet. Gynecol. Reprod. Biol..

[7] Dumollard R., Duchen M., Carroll J. (2007). The role of mitochondrial function in the oocyte and embryo. Curr. Top. Dev. Biol..

[8] James A.M., Sharpley M.S., Manas A.R., Frerman F.E., Hirst J., Smith R.A., Murphy M.P. (2007). Interaction of the mitochondria-targeted antioxidant MitoQ with phospholipid bilayers and ubiquinone oxidoreductases. J. Biol. Chem..

[9] Kelso G.F., Porteous C.M., Coulter C.V., Hughes G., Porteous W.K., Ledgerwood E.C., Smith R.A., Murphy M.P. (2001). Selective targeting of a redox-active ubiquinone to mitochondria within cells: Antioxidant and antiapoptotic properties. J. Biol. Chem..

[10] James A.M., Cocheme H.M., Smith R.A., Murphy M.P. (2005). Interactions of mitochondria-targeted and untargeted ubiquinones with the mitochondrial respiratory chain and reactive oxygen species. Implications for the use of exogenous ubiquinones as therapies and experimental tools. J. Biol. Chem..

[11] Ramsey H., Zhang Q., Wu M.X. (2015). Mitoquinone restores platelet production in irradiation-induced thrombocytopenia. Platelets.

[12] Gane E.J., Weilert F., Orr D.W., Keogh G.F., Gibson M., Lockhart M.M., Frampton C.M., Taylor K.M., Smith R.A., Murphy M.P. (2010). The mitochondria-targeted anti-oxidant mitoquinone decreases liver damage in a phase II study of hepatitis C patients. Liver. Int..

[13] Snow B.J., Rolfe F.L., Lockhart M.M., Frampton C.M., O’Sullivan J.D., Fung V., Smith R.A., Murphy M.P., Taylor K.M., Protect Study G. (2010). A double-blind, placebo-controlled study to assess the mitochondria-targeted antioxidant MitoQ as a disease-modifying therapy in Parkinson’s disease. Mov. Disord..

[14] Rodriguez-Cuenca S., Cocheme H.M., Logan A., Abakumova I., Prime T.A., Rose C., Vidal-Puig A., Smith A.C., Rubinsztein D.C., Fearnley I.M. (2010). Consequences of long-term oral administration of the mitochondria-targeted antioxidant MitoQ to wild-type mice. Free Radic. Biol. Med..

[15] Al-Zubaidi U., Adhikari D., Cinar O., Zhang Q.H., Yuen W.S., Murphy M.P., Rombauts L., Robker R.L., Carroll J. (2021). Mitochondria-targeted therapeutics, MitoQ and BGP-15, reverse aging-associated meiotic spindle defects in mouse and human oocytes. Hum. Reprod..

[16] Zhou D., Zhuan Q., Luo Y., Liu H., Meng L., Du X., Wu G., Hou Y., Li J., Fu X. (2022). Mito-Q promotes porcine oocytes maturation by maintaining mitochondrial thermogenesis via UCP2 downregulation. Theriogenology.

[17] Zou K., Hou L., Sun K., Xie W., Wu J. (2011). Improved efficiency of female germline stem cell purification using fragilis-based magnetic bead sorting. Stem Cells Dev..

[18] Hamazaki N., Kyogoku H., Araki H., Miura F., Horikawa C., Hamada N., Shimamoto S., Hikabe O., Nakashima K., Kitajima T.S. (2021). Reconstitution of the oocyte transcriptional network with transcription factors. Nature.

[19] Green D.R., Reed J.C. (1998). Mitochondria and apoptosis. Science.

[20] Cao L., Shitara H., Sugimoto M., Hayashi J., Abe K., Yonekawa H. (2009). New evidence confirms that the mitochondrial bottleneck is generated without reduction of mitochondrial DNA content in early primordial germ cells of mice. PLoS Genet..

[21] Lei L., Spradling A.C. (2016). Mouse oocytes differentiate through organelle enrichment from sister cyst germ cells. Science.

[22] Cao L., Shitara H., Horii T., Nagao Y., Imai H., Abe K., Hara T., Hayashi J., Yonekawa H. (2007). The mitochondrial bottleneck occurs without reduction of mtDNA content in female mouse germ cells. Nat. Genet..

[23] Dalton C.M., Szabadkai G., Carroll J. (2014). Measurement of ATP in single oocytes: Impact of maturation and cumulus cells on levels and consumption. J. Cell. Physiol..

[24] Zhang X., Wu X.Q., Lu S., Guo Y.L., Ma X. (2006). Deficit of mitochondria-derived ATP during oxidative stress impairs mouse MII oocyte spindles. Cell Res..

[25] Wu L.L., Russell D.L., Wong S.L., Chen M., Tsai T.S., St John J.C., Norman R.J., Febbraio M.A., Carroll J., Robker R.L. (2015). Mitochondrial dysfunction in oocytes of obese mothers: Transmission to offspring and reversal by pharmacological endoplasmic reticulum stress inhibitors. Development.

[26] Oyewole A.O., Birch-Machin M.A. (2015). Mitochondria-targeted antioxidants. FASEB J..

[27] Schafer M., Farwanah H., Willrodt A.H., Huebner A.J., Sandhoff K., Roop D., Hohl D., Bloch W., Werner S. (2012). Nrf2 links epidermal barrier function with antioxidant defense. EMBO Mol. Med..

[28] Meewes C., Brenneisen P., Wenk J., Kuhr L., Ma W., Alikoski J., Poswig A., Krieg T., Scharffetter-Kochanek K. (2001). Adaptive antioxidant response protects dermal fibroblasts from UVA-induced phototoxicity. Free Radic. Biol. Med..

[29] Zhu R., Chen B., Bai Y., Miao T., Rui L., Zhang H., Xia B., Li Y., Gao S., Wang X.D. (2020). Lycopene in protection against obesity and diabetes: A mechanistic review. Pharmacol. Res..

[30] You J., Kim J., Lim J., Lee E. (2010). Anthocyanin stimulates in vitro development of cloned pig embryos by increasing the intracellular glutathione level and inhibiting reactive oxygen species. Theriogenology.

[31] Zhang X., Zhou C., Li W., Li J., Wu W., Tao J., Liu H. (2020). Vitamin C Protects Porcine Oocytes From Microcystin-LR Toxicity During Maturation. Front. Cell Dev. Biol..

[32] Liu C., Shui S., Yao Y., Sui C., Zhang H. (2020). Ascorbic acid ameliorates dysregulated folliculogenesis induced by mono-(2-ethylhexyl)phthalate in neonatal mouse ovaries via reducing ovarian oxidative stress. Reprod. Domest. Anim..

[33] Endo T., Mikedis M.M., Nicholls P.K., Page D.C., de Rooij D.G. (2019). Retinoic Acid and Germ Cell Development in the Ovary and Testis. Biomolecules.

[34] Jauslin M.L., Meier T., Smith R.A., Murphy M.P. (2003). Mitochondria-targeted antioxidants protect Friedreich Ataxia fibroblasts from endogenous oxidative stress more effectively than untargeted antioxidants. FASEB J..

[35] Genrikhs E.E., Stelmashook E.V., Popova O.V., Kapay N.A., Korshunova G.A., Sumbatyan N.V., Skrebitsky V.G., Skulachev V.P., Isaev N.K. (2015). Mitochondria-targeted antioxidant SkQT1 decreases trauma-induced neurological deficit in rat and prevents amyloid-beta-induced impairment of long-term potentiation in rat hippocampal slices. J. Drug Target..

[36] Gioscia-Ryan R.A., LaRocca T.J., Sindler A.L., Zigler M.C., Murphy M.P., Seals D.R. (2014). Mitochondria-targeted antioxidant (MitoQ) ameliorates age-related arterial endothelial dysfunction in mice. J. Physiol..

[37] Supinski G.S., Murphy M.P., Callahan L.A. (2009). MitoQ administration prevents endotoxin-induced cardiac dysfunction. Am. J. Physiol. Regul. Integr. Comp. Physiol..

[38] Dashdorj A., Jyothi K.R., Lim S., Jo A., Nguyen M.N., Ha J., Yoon K.S., Kim H.J., Park J.H., Murphy M.P. (2013). Mitochondria-targeted antioxidant MitoQ ameliorates experimental mouse colitis by suppressing NLRP3 inflammasome-mediated inflammatory cytokines. BMC Med..

[39] Oyewole A.O., Wilmot M.C., Fowler M., Birch-Machin M.A. (2014). Comparing the effects of mitochondrial targeted and localized antioxidants with cellular antioxidants in human skin cells exposed to UVA and hydrogen peroxide. FASEB J..

[40] Saretzki G., Murphy M.P., von Zglinicki T. (2003). MitoQ counteracts telomere shortening and elongates lifespan of fibroblasts under mild oxidative stress. Aging Cell.

[41] Graham D., Huynh N.N., Hamilton C.A., Beattie E., Smith R.A., Cocheme H.M., Murphy M.P., Dominiczak A.F. (2009). Mitochondria-targeted antioxidant MitoQ10 improves endothelial function and attenuates cardiac hypertrophy. Hypertension.

[42] Dumollard R., Campbell K., Halet G., Carroll J., Swann K. (2008). Regulation of cytosolic and mitochondrial ATP levels in mouse eggs and zygotes. Dev. Biol..

[43] Johnson M.T., Freeman E.A., Gardner D.K., Hunt P.A. (2007). Oxidative metabolism of pyruvate is required for meiotic maturation of murine oocytes in vivo. Biol. Reprod..

[44] Rasmussen L.M., Sen N., Vera J.C., Liu X., Craig Z.R. (2017). Effects of in vitro exposure to dibutyl phthalate, mono-butyl phthalate, and acetyl tributyl citrate on ovarian antral follicle growth and viability. Biol. Reprod..

[45] Smitz J.E., Cortvrindt R.G. (2002). The earliest stages of folliculogenesis in vitro. Reproduction.

[46] Silva G.M., Brito I.R., Sales A.D., Aguiar F.L.N., Duarte A.B.G., Araujo V.R., Vieira L.A., Magalhaes-Padilha D.M., Lima L.F., Alves B.G. (2017). In vitro growth and maturation of isolated caprine preantral follicles: Influence of insulin and FSH concentration, culture dish, coculture, and oocyte size on meiotic resumption. Theriogenology.

[47] Magalhaes D.M., Duarte A.B., Araujo V.R., Brito I.R., Soares T.G., Lima I.M., Lopes C.A., Campello C.C., Rodrigues A.P., Figueiredo J.R. (2011). In vitro production of a caprine embryo from a preantral follicle cultured in media supplemented with growth hormone. Theriogenology.

[48] Passos M.J., Vasconcelos G.L., Silva A.W., Brito I.R., Saraiva M.V., Magalhaes D.M., Costa J.J., Donato M.A., Ribeiro R.P., Cunha E.V. (2013). Accelerated growth of bovine preantral follicles in vitro after stimulation with both FSH and BMP-15 is accompanied by ultrastructural changes and increased atresia. Theriogenology.

[49] Xu J., Lawson M.S., Yeoman R.R., Molskness T.A., Ting A.Y., Stouffer R.L., Zelinski M.B. (2013). Fibrin promotes development and function of macaque primary follicles during encapsulated three-dimensional culture. Hum. Reprod..

[50] Xu J., Bernuci M.P., Lawson M.S., Yeoman R.R., Fisher T.E., Zelinski M.B., Stouffer R.L. (2010). Survival, growth, and maturation of secondary follicles from prepubertal, young, and older adult rhesus monkeys during encapsulated three-dimensional culture: Effects of gonadotropins and insulin. Reproduction.

[51] Spears N., Boland N.I., Murray A.A., Gosden R.G. (1994). Mouse oocytes derived from in vitro grown primary ovarian follicles are fertile. Hum. Reprod..

[52] Xu J., Zelinski M.B. (2022). Oocyte quality following in vitro follicle developmentdagger. Biol. Reprod..

[53] Secomandi L., Borghesan M., Velarde M., Demaria M. (2022). The role of cellular senescence in female reproductive aging and the potential for senotherapeutic interventions. Hum. Reprod. Update.

[54] Lu X., Liu Y., Xu J., Cao X., Zhang D., Liu M., Liu S., Dong X., Shi H. (2022). Mitochondrial dysfunction in cumulus cells is related to decreased reproductive capacity in advanced-age women. Fertil. Steril..

[55] Kim S., Song J., Ernst P., Latimer M.N., Ha C.M., Goh K.Y., Ma W., Rajasekaran N.S., Zhang J., Liu X. (2020). MitoQ regulates redox-related noncoding RNAs to preserve mitochondrial network integrity in pressure-overload heart failure. Am. J. Physiol. Heart Circ. Physiol..

[56] Ojano-Dirain C.P., Antonelli P.J., Le Prell C.G. (2014). Mitochondria-targeted antioxidant MitoQ reduces gentamicin-induced ototoxicity. Otol. Neurotol..

[57] Landry D.A., Yakubovich E., Cook D.P., Fasih S., Upham J., Vanderhyden B.C. (2022). Metformin prevents age-associated ovarian fibrosis by modulating the immune landscape in female mice. Sci. Adv..

[58] Sheng X., Tian C., Liu L., Wang L., Ye X., Li J., Zeng M., Liu L. (2019). Characterization of oogonia stem cells in mice by Fragilis. Protein Cell.

[59] Wu C., Xu B., Li X., Ma W., Zhang P., Chen X., Wu J. (2017). Tracing and Characterizing the Development of Transplanted Female Germline Stem Cells In Vivo. Mol. Ther..

[60] Fan H.Y., Liu Z., Shimada M., Sterneck E., Johnson P.F., Hedrick S.M., Richards J.S. (2009). MAPK3/1 (ERK1/2) in ovarian granulosa cells are essential for female fertility. Science.

[61] Fan H.Y., Liu Z., Johnson P.F., Richards J.S. (2011). CCAAT/enhancer-binding proteins (C/EBP)-alpha and -beta are essential for ovulation, luteinization, and the expression of key target genes. Mol. Endocrinol..

